# Cytogenetic risk–associated outcomes of antithymocyte globulin use in HLA-matched sibling transplantation for acute myeloid leukemia

**DOI:** 10.1007/s00277-025-06608-3

**Published:** 2025-10-16

**Authors:** Mihee Kim, Ik-Chan Song, Seo-Yeon Ahn, Ho Cheol Jang, Jeong Suk Koh, Chang-Hoon Lee, Hyeoung-Joon Kim, Ho-Young Yhim, Jae-Sook Ahn

**Affiliations:** 1 Hematology-Oncology, Chonnam National University Hwasun Hospital, Hwasun-gun, Jeollanam-do Republic of Korea; 2 Genomic Research Center for Hematopoietic Diseases, Chonnam National University Hwasun Hospital, Hwasun-gun, Jeollanam-do Republic of Korea; 3 Division of Hematology/Oncology, Department of Internal Medicine, Chungnam National University College of Medicine, Daejeon, Republic of Korea; 4 Hematology-Oncology, Department of Internal Medicine, Jeonbuk National University Medical School, Jeonju, Republic of Korea; 5 Department of Internal Medicine, Jeonbuk National University Medical School, 20, Geonjiro Deokjin-gu, Jeonju-si, Jeollabuk-do 54907 Republic of Korea; 6 Department of Internal Medicine, Chonnam National University Hwasun Hospital, Chonnam National University, 322 Seoyang-ro, Hwasun-eup, , Hwasun-gun, Jeollanam-do 58128 Republic of Korea

**Keywords:** Acute myeloid leukemia, Antithymocyte globulin, Graft-versus-host disease, Chronic graft-versus-host disease-free relapse-free survival

## Abstract

Antithymocyte globulin (ATG) reduces chronic graft-versus-host disease (cGvHD), but concerns remain regarding relapse risk, particularly across cytogenetic risk groups. We retrospectively analyzed 148 acute myeloid leukemia (AML) patients in first remission who underwent matched sibling allogeneic hematopoietic stem cell transplantation (HSCT) across three centers. Patients received transplantation with or without ATG (2.5–5 mg/kg) and were stratified by cytogenetic risk (intermediate, *n* = 128; adverse, *n* = 20). Propensity score matching and inverse probability of treatment weighting were applied to adjust for baseline imbalances. After a median follow-up of 91 months, ATG use (*n* = 96) reduced the incidence of cGvHD (HR 0.43; 95% CI 0.26–0.72; *p* = 0.001) and improved cGvHD-free relapse-free survival (cGRFS; HR 0.61; 95% CI 0.40–0.93; *p* = 0.022). No significant differences were observed in overall survival, event-free survival (EFS), relapse, or non-relapse mortality. A significant interaction between ATG and cytogenetic risk (*p* = 0.025) indicated differential effects by risk profiles. In subgroup analyses, intermediate-risk patients derived consistent benefit, whereas adverse-risk patients (ATG *n* = 12; non-ATG *n* = 8) showed higher relapse and inferior EFS. With respect to dose, both 2.5 and 5 mg/kg reduced cGvHD relative to non-ATG; exploratory analyses suggested that 2.5 mg/kg may provide adequate prophylaxis without excess relapse, although statistical power was limited. In conclusion, ATG was associated with reduced cGvHD and improved cGRFS in HSCT for AML, with benefit mainly in intermediate-risk patients. Its use in adverse-risk AML warrants caution, as it may be associated with higher relapse.

**Supplementary Information:**

The online version contains supplementary material available at 10.1007/s00277-025-06608-3.

## Introduction

Allogeneic hematopoietic stem cell transplantation (allo-HSCT) is regarded as optimal curative approach for patients with acute myeloid leukemia (AML) [1, 2]. Despite significant advances in transplantation techniques, relapse and graft-versus-host disease (GvHD) remain major challenges after allo-HSCT, substantially affecting both long-term survival and quality of life [3, 4]. Antithymocyte globulin (ATG) has been incorporated into conditioning regimens to mitigate GvHD, and its efficacy in reducing the incidence and severity of GvHD is well established [5–7]. By depleting T lymphocytes, ATG reduces alloreactivity and the risk of GvHD. At the same time, this immunosuppressive effect may compromise the graft-versus-leukemia (GVL) effect, raising concerns about increased relapse risk [5–7].

A critical consideration in ATG administration is the underlying disease characteristics of each patient, particularly cytogenetic risk status. High-risk cytogenetics have consistently been associated with increased relapse rates regardless of GvHD prophylaxis strategies, suggesting the need for more nuanced approaches to ATG administration [7, 8]. The relationship between ATG dosing and chronic GvHD (cGvHD)-free, relapse-free survival (cGRFS) has emerged as an integrated endpoint to evaluate both GvHD prevention and disease control [7, 9, 10]. Some studies have suggested that the clinical impact of ATG may differ according to cytogenetic context, with potential benefit in non-adverse cytogenetic risk groups and unfavorable effects in adverse-risk patients [11]. However, systematic evaluation in AML patients undergoing HLA-matched sibling transplantation is lacking.

The optimal dosing strategy for ATG remains a subject of ongoing investigation, particularly in the context of HLA-matched sibling donor (MSD) transplantation. Early studies have shown conflicting results regarding the impact of ATG on transplant outcomes [5, 8, 11–14]. The Center for International Blood and Marrow Transplant Research (CIBMTR) analysis suggested the potential risk of relapse associated with in vivo T-cell depletion (median 7 mg/kg) [5]; meanwhile, the European Society for Blood and Marrow Transplantation (EBMT) data demonstrated ATG (median 5 mg/kg) reduced the incidence of GvHD without increasing the risk of relapse in AML patients undergoing allo-HSCT from an MSD [13]. These divergent findings have sparked renewed interest in dose-dependent effects [11, 14, 15]. This has led to a general categorization in the literature of high-dose (beyond 6 mg/kg) versus low-dose (2.5–6 mg/kg) ATG. However, the optimal dosing strategy within these ranges, particularly for MSD transplantation, remains to be established [7, 16]. Moreover, beyond absolute dosing, recent studies indicated that clinical outcomes may also depend on the degree of ATG exposure, which is influenced by individual characteristics such as lymphocyte count and drug clearance [17–20].

Against this background, we conducted a multi-center retrospective study to evaluate the impact of ATG on outcomes in AML patients undergoing allo-HSCT from HLA-matched sibling donors, with particular attention to differences across cytogenetic risk groups. Exploratory analyses were also performed to compare outcomes between ATG doses of 2.5 mg/kg and 5 mg/kg.

## Materials and methods

### Patients and study design

This retrospective study included adult patients with AML who underwent allo-HSCT from HLA–MSD between May 2003 and December 2022 at three institutions. An MSD was defined as an 8/8 HLA allele match at HLA-A, -B, -C, and DRB1 loci between donor and recipient. Eligible patients were aged ≥ 18 years, were in first complete remission (CR1) at the time of transplantation, and received peripheral blood stem cells (PBSCs) as the graft source. Since this study was retrospective, ATG use and doses (2.5 mg/kg or 5.0 mg/kg) varied by institutional practice. ATG administration was generally initiated following the inclusion of ATG in the Korean National Health Insurance reimbursement program; dosing strategies followed the clinical protocols from each center.

Cytogenetic risk was classified according to the Medical Research Council (MRC) criteria [21], and only patients with intermediate or adverse risk were included in the analysis. Patients were excluded if they had acute promyelocytic leukemia (APL), active disease at the time of transplantation, received cord blood or bone marrow as the graft source, or had a history of prior allo-HSCT. Patients with favorable MRC cytogenetic risk were also excluded.

For patients with available next-generation sequencing (NGS) data, additional risk stratification was performed according to the European LeukemiaNet (ELN) 2022 criteria for subgroup analyses [22]. Chronic GvHD (cGvHD) was classified as limited or extensive according to the Seattle criteria. The Institutional Review Boards approved the study protocol of all participating centers.

### Conditioning regimen and GvHD prophylaxis

Myeloablative conditioning (MAC) consisted of busulfan (12.8 mg/kg intravenously) plus cyclophosphamide (120 mg/kg intravenously) or fludarabine (150 mg/m² intravenously) plus busulfan (12.8 mg/kg intravenously). Reduced-intensity conditioning (RIC) included fludarabine (150 mg/m² intravenously) plus busulfan (6.4 mg/kg intravenously) or fludarabine (150 mg/m² intravenously) plus melphalan (140 mg/m² intravenously). GvHD prophylaxis consisted of cyclosporine and methotrexate (15 mg/m² on day + 1 and 10 mg/m² on days + 3, +6, and + 11). Patients in the ATG group received rabbit ATG (Thymoglobulin^®^) either at a dose of 2.5 mg/kg or 5.0 mg/kg, administered over 2–3 days, with the last dose administered on day − 2 or −1 before stem cell infusion. Cyclosporine was initiated at 3 mg/kg/day intravenously from day − 1 and adjusted to maintain blood levels.

### Statistical analysis

Patient characteristics were compared using the chi-square test for categorical variables and the Mann–Whitney U test for continuous variables. Continuous variables (age, infused CD34⁺ cell dose, HCT-CI, and total T-cell count) were categorized using either cohort medians or clinically relevant cut-offs (age ≥ 48 years, HCT-CI ≥ 3, CD34⁺ ≥4.0 × 10⁶/kg, T-cell count ≥ 29.5 × 10⁷/kg). Overall survival (OS) was calculated from the date of transplantation until death from any cause. Event-free survival (EFS) was defined as survival without relapse or death. cGRFS was defined as survival without relapse, death, or cGvHD. Non-relapse mortality (NRM) was defined as death without prior relapse. Relapse was defined by morphological evidence of disease in bone marrow, peripheral blood, or extramedullary sites. For the competing risk analysis of cGvHD, relapse, and NRM, we used the cumulative incidence method with death from other causes as the competing event. Specifically, for GvHD analysis, death without GvHD was treated as a competing risk. For relapse analysis, death without relapse was considered as a competing event, and for NRM analysis, relapse was treated as a competing event. The differences in cumulative incidences between groups were compared using Gray’s test.

Survival probabilities for OS, EFS, and cGRFS were estimated by the Kaplan–Meier method and compared using the log-rank test. To adjust for baseline imbalances, propensity scores were estimated using a multinomial logistic regression model including infused CD34⁺ cell dose (≥ 4.0 × 10⁶/kg vs. < 4.0 × 10⁶/kg), conditioning regimen (myeloablative [MAC] vs. reduced-intensity [RIC]), cytogenetic risk (intermediate vs. adverse), and Hematopoietic Cell Transplantation–Comorbidity Index (HCT-CI; ≥ 3 vs. < 3). Inverse probability of treatment weighting (IPTW) with stabilization and truncation was applied, and propensity score matching was performed as a sensitivity analysis. Weighted Cox proportional hazards models were used for OS, EFS, and cGRFS, and weighted Fine–Gray models were applied for cGvHD, relapse, and NRM. Adjusted hazard ratios (HRs) were visualized in forest plots.

To examine whether the effect of ATG varied across subgroups, we included multiplicative interaction terms between ATG use and clinical covariates in the Cox and Fine–Gray models. Interactions were tested for cytogenetic risk (intermediate vs. adverse), HCT-CI (< 3 vs. ≥ 3), and conditioning intensity (MAC vs. RIC).

Given the multi-center nature of this study, the transplant center was included as a covariate in the univariate analysis to adjust for potential institutional variability in clinical practice. Variables with a *p*-value < 0.1 in the univariate analysis were included in the multivariate model. All statistical analyses were performed using EZR version 1.55; R version 4.3.2. The *p*-values < 0.05 were considered statistically significant [23].

## Results

### Characteristics of patients

A total of 148 AML patients who underwent allo-HSCT from an HLA-matched sibling were included. Of these, 58 (39.2%) received ATG and 90 (60.8%) did not. Baseline characteristics were largely comparable between groups in terms of age (≥ 48 years; 65.5% vs. 53.3%; *p* = 0.173) and time from diagnosis to transplantation (4.8 vs. 4.1 months; *p* = 0.484). The ATG group had a higher proportion of patients with adverse cytogenetics (20.7% vs. 8.9%). High HCT-CI (≥ 3; 6.9% vs. 1.1%; *p* = 0.077) and RIC use (25.9% vs. 11.2%; *p* = 0.025) were also more common with ATG, while high CD34⁺ cell dose was less frequent (≥ 4.0 × 10^6^/kg, 41.4% vs. 58.9%; *p* = 0.044). Donor characteristics were similar (Table 1).

**Table 1:** Baseline characteristics of patients according to antithymocyte Globulin (ATG) administration

Characteristics	Non-ATG group (*n* = 90)	ATG group (*n* = 58)	*p*-value
ATG dose, median (range)	-	5 (2.5–5)	-
Age at HSCT, years, median (range)	46.0 (22–65)	53.0 (18–70)	
≥ 48	48 (53.3)	38 (65.5)	0.173
< 48	42 (46.7)	20 (34.5)	
Time from diagnosis to HSCT, months	4.1 (2.8–8.8)	4.8 (2.9–19.3)	0.484
Sex, male	44 (48.9)	30 (51.7)	0.866
Cytogenetic risk			0.050
Intermediate risk	82 (91.1)	46 (79.3)	
Adverse risk	8 (8.9)	12 (20.7)	
ELN-2022 risk stratification	*n* = 55	*n* = 55	0.010
Favorable risk	12 (21.8)	2 (3.6)	
Intermediate risk	28 (50.9)	37 (67.3)	
Adverse risk	15 (27.3)	16 (29.1)	
HCT-CI score			0.041 ^a^
0	80 (88.9)	43 (74.1)	0.077 ^b^
1–2	9 (10.0)	11 (19.0)	
≥ 3	1 (1.1)	4 (6.9)	
Conditioning regimen			0.025
MAC	79 (88.8)	43 (74.1)	
RIC	10 (11.2)	15 (25.9)	
ABO matched donor	52 (57.8)	36 (62.1)	0.732
Sex matched donor	54 (60.0)	27 (46.6)	0.129
Infused CD34 + cell dose (median, x 10^6^/kg)	4.5 (0.6–20.0)	3.6 (0.9–13.4)	
≥ 4.0	53 (58.9)	24 (41.4)	0.044
< 4.0	37 (41.1)	34 (58.6)	
Total T cell count (median, x 10^7^/kg)	30.2 (2.3–129.5)	29.1 (13.5–56.3)	
≥ 29.5	43 (47.8)	21 (36.2)	0.104
< 29.5	47 (52.2)	37 (63.8)	
ALC on the day of ATG initiation (median, x 10^9^/L)	*n* = 56 0.05 (0-1.10)	*n* = 46 0.02 (0-0.64)	
> 0.04	33 (58.9)	12 (26.1)	0.001
≤ 0.04	23 (41.1)	34 (73.9)	
Duration of follow-up, months	117.1 (22.8–189.7)	49.3 (5.2–100.3)	< 0.001

Values are presented as number (%) or median (range), as appropriate

^a^ Overall comparison across the three groups

^b^ Comparison between HCT-CI ≥ 3 vs. < 3

Before adjustment, significant imbalances were observed between groups in terms of cytogenetic risk, infused CD34⁺ cell dose, and conditioning regimen. After IPTW adjustment, baseline characteristics were well balanced across treatment groups (all standardized mean differences < 0.1; Supplementary Table 1). The median follow-up of survivors was 91.0 months. The 5-year OS, EFS, and cGRFS for the entire cohort were 53.9%, 51.3%, and 18.8%, respectively (Supplementary Fig. 1). F.

### Overall transplant outcomes: ATG vs. non-ATG

**Fig. 1:**
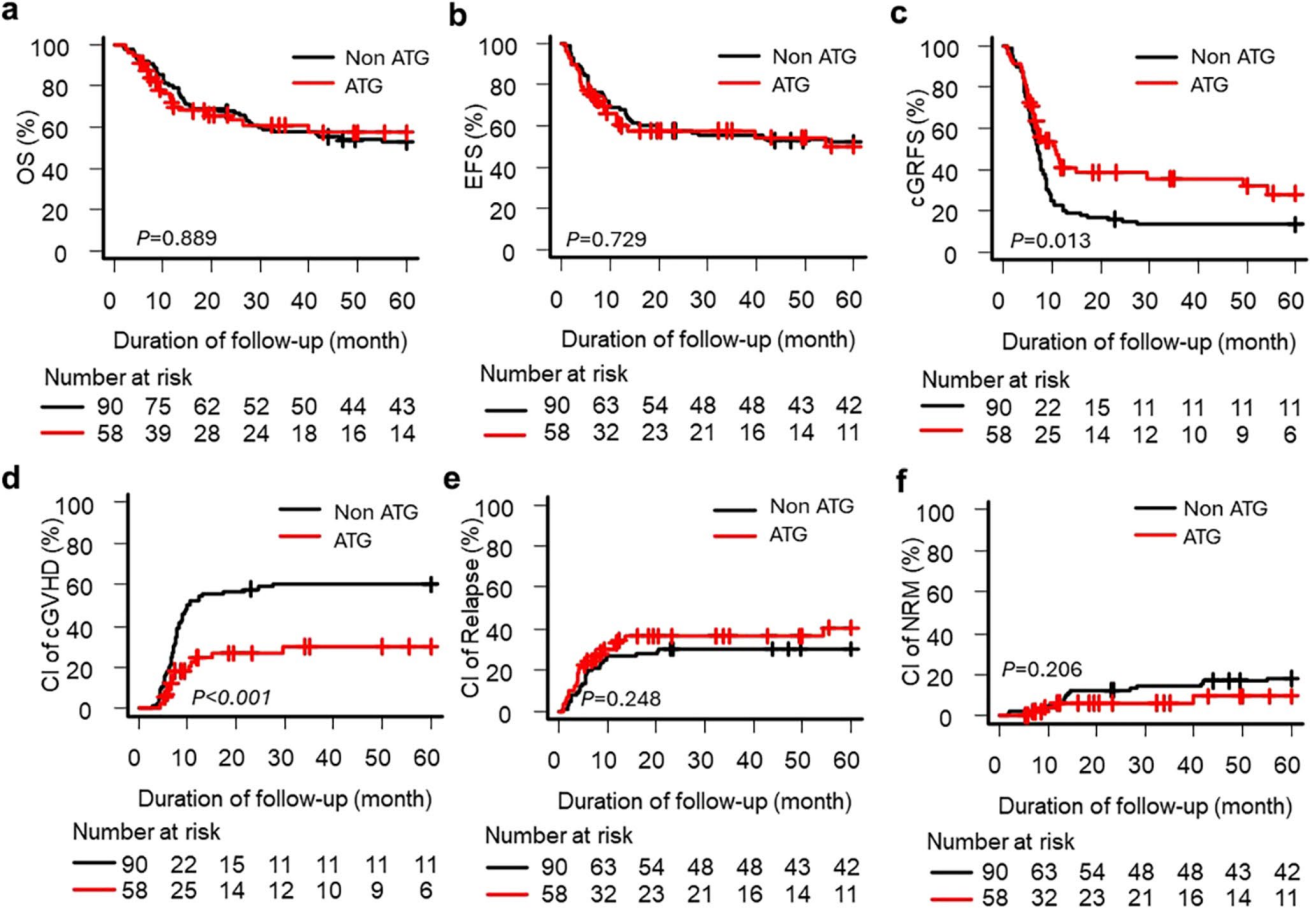
Transplantation outcomes in the entire cohort according to antithymocyte globulin (ATG) administration (**a**) Overall survival (OS), (**b**) event-free survival (EFS), (**c**) chronic graft-versus-host disease (cGvHD)-free relapse-free survival (cGRFS), (**d**) cumulative incidence (CI) of cGvHD, (**e**) cumulative incidence of relapse, and (**f**) cumulative incidence of non-relapse mortality (NRM). Adjusted hazard ratios are presented in Supplementary Fig. 2

ATG use did not influence OS or EFS but was associated with superior 5-year cGRFS (28.0% vs. 13.2%; *p* = 0.013) and a markedly lower incidence of cGvHD (29.8% vs. 60.2%; *p* < 0.001), including extensive cGvHD (10.3% vs. 37.8%; *p* < 0.001; Table 2; Fig. 1). Relapse incidence and NRM did not differ between groups. IPTW analysis confirmed a significant reduction in cGvHD (HR 0.43; 95% Confidence Interval [CI] 0.26–0.62; *p* = 0.001) and improved cGRFS (HR 0.61; 95% CI 0.40–0.93; *p* = 0.022), with no impact on OS, EFS, relapse, or NRM (Supplementary Fig. 2).

**Table 2:** Analysis of chronic GvHD (cGvHD), relapse, and non-relapse mortality by ATG groups

*n* (%)	Non-ATG (*n* = 90)	ATG: 2.5 mg/kg (*n* = 17)	ATG: 5 mg/kg (*n* = 41)	*p*-value
cGvHD	54 (60.0)	5 (29.4)	10 (24.4)	< 0.001^a^; < 0.001^b^; 0.438 ^c^
Onset (median, months)	6.9	10.2	7.1	0.879
Organ involvement, n (%)				0.114
Skin	24 (26.7)	2 (11.8)	6 (14.6)	
GI tract	7 (7.8)	0	2 (4.9)	
Liver	23 (25.6)	1 (5.9)	10 (24.4)	
Ocular	9 (10.0)	2 (11.8)	1 (2.4)	
Pulmonary	11 (12.2)	1 (5.9)	4 (9.8)	
Oral mucosa	15 (16.7)	3 (17.6)	2 (4.9)	
Severity of cGVHD, n (%)				0.001^a^; 0.001^b^; 0.241^c^
Limited	20 (22.2)	3 (17.6)	6 (14.6)	
Extensive	34 (37.8)	2 (11.8)	4 (9.8)	
Relapse, n (%)	28 (31.1)	4 (23.5)	17 (41.5)	0.340^a^; 0.520 ^b^; 0.196^c^
Non-relapse mortality, n (%)	16 (17.8)	0	4 (9.8)	0.103 ^a^ 0.059 ^b^; 0.182 ^c^

Unless otherwise indicated, values are presented as number (%) or median (range)

^a^ Overall comparison across the three groups

^b^ Comparison between ATG and. non-ATG groups

^c^ Comparison between 2.5 mg/kg ATG and 5 mg/kg ATG groups

Abbreviations: *ATG* antithymocyte globulin, *cGvHD* chronic graft-versus-host disease, *GI* gastrointestinal

### Outcomes by cytogenetic and ELN risk

**Fig. 2:**
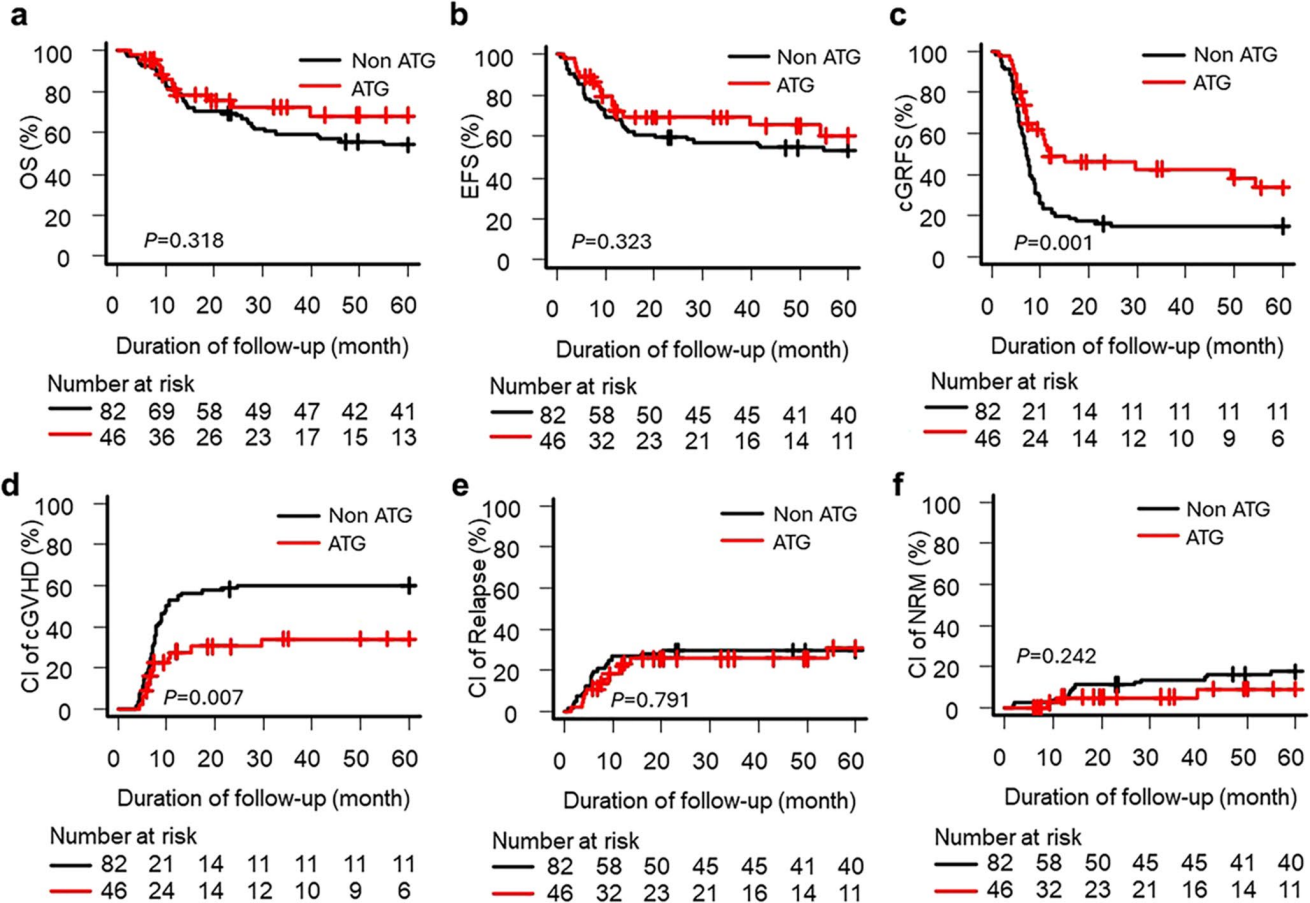
Transplantation outcomes in patients with intermediate cytogenetics, stratified by antithymocyte globulin (ATG) administration (**a**) Overall survival (OS), (**b**) event-free survival (EFS), (**c**) chronic graft-versus-host disease (cGvHD)-free relapse-free survival (cGRFS), (**d**) cumulative incidence (CI) of cGvHD, (**e**) cumulative incidence of relapse, and (**f**) cumulative incidence of non-relapse mortality (NRM). Adjusted hazard ratios are presented in Supplementary Fig. 2

**Fig. 3:**
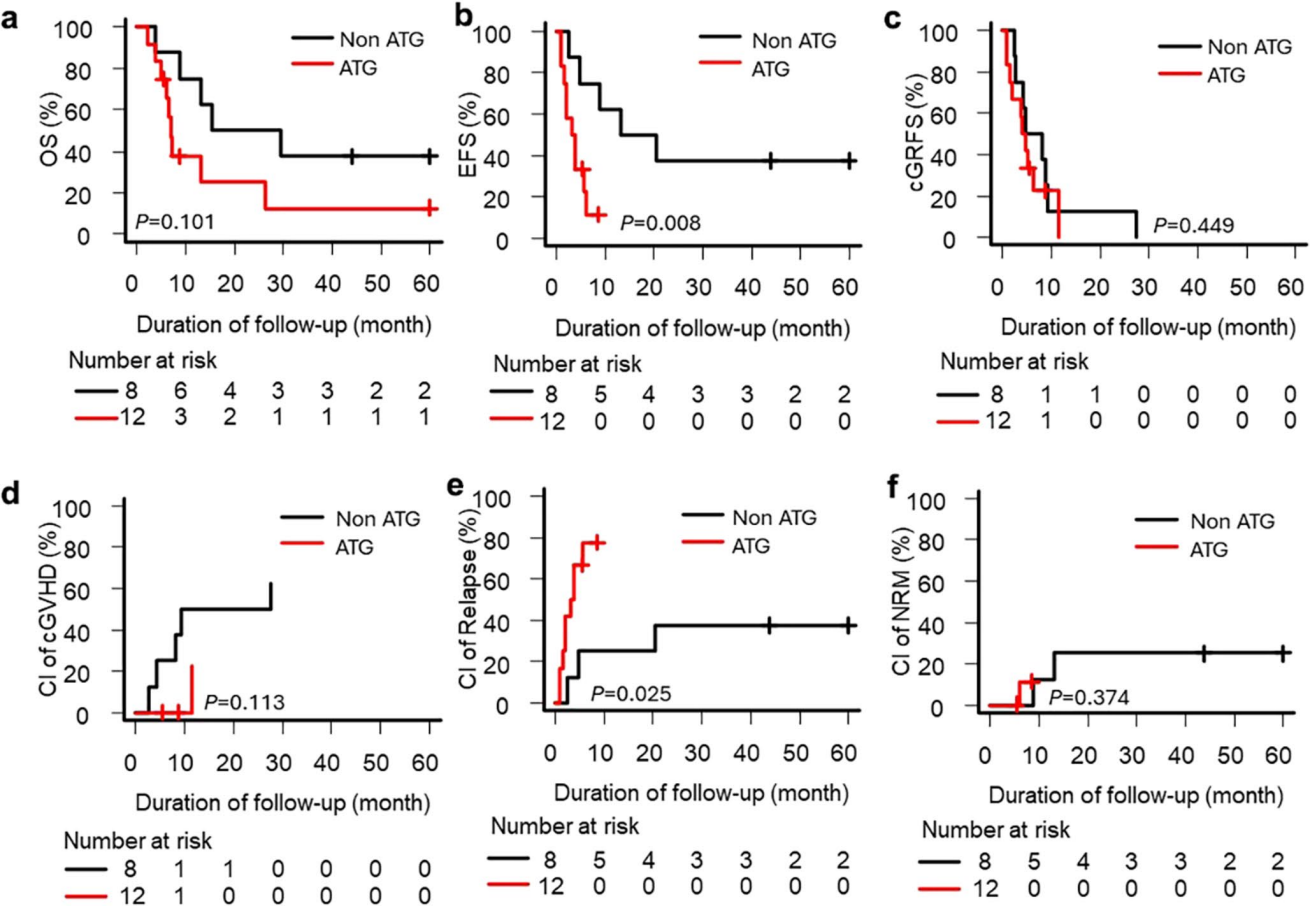
Transplantation outcomes in patients with adverse cytogenetics, stratified by antithymocyte globulin (ATG) administration (**a**) Overall survival (OS), (**b**) event-free survival (EFS), (**c**) chronic graft-versus-host disease (cGvHD)-free relapse-free survival (cGRFS), (**d**) cumulative incidence (CI) of cGvHD, (**e**) cumulative incidence of relapse, and (**f**) cumulative incidence of non-relapse mortality (NRM). Adjusted hazard ratios are presented in Supplementary Fig. 2

Of 148 patients, 128 (86.4%) had intermediate-risk and 20 (13.5%) adverse-risk cytogenetics. ATG use was more frequent in the adverse group (60.0% vs. 35.9%; *p* = 0.050; Table 1). In the intermediate-risk patients, ATG significantly improved cGRFS (5-year, 33.6% vs. 14.5%; *p* = 0.001) and reduced cGvHD (33.9% vs. 59.9%; *p* = 0.007), with no effect on OS, EFS, relapse, or NRM (Fig. 2). IPTW analysis confirmed these associations. (Supplementary Fig. 2). Conversely, in the adverse-risk group, ATG was associated with inferior EFS (*p* = 0.008) and higher relapse risk (*p* = 0.025) without effect on OS, cGRFS, cGvHD, or NRM (Fig. 3a–f). IPTW analysis confirmed the higher relapse risk in the ATG group (HR 4.88; 95% CI 1.52–15.68; *p* = 0.007) and no protective effect on cGvHD or cGRFS (Supplementary Fig. 2).

The ELN-2022 risk classification was available for 110 patients. Among ELN-intermediate patients, ATG reduced cGvHD (*p* = 0.005) and improved cGRFS (*p* = 0.005), with no significant impact on OS, EFS, relapse, or NRM (Supplementary Fig. 3). These findings remained consistent after IPTW adjustment. In the ELN-adverse group, ATG significantly reduced cGvHD (*p* = 0.008) but was associated with higher relapse risk (*p* = 0.045) and inferior EFS (*p* = 0.010) without affecting OS, NRM, or cGRFS (Supplementary Fig. 4). After IPTW weighting, the results were similar, with ATG showing only a trend toward inferior EFS (*p* = 0.09) and increased relapse risk (*p* = 0.08), which did not reach statistical significance.

### Impact of ATG dose

Among ATG recipients (*n* = 58), 17 patients received 2.5 mg/kg, and 41 received 5 mg/kg. Baseline characteristics were similar except for a higher HCT-CI ≥ 3 and infused CD34⁺ cell doses in the 2.5 mg/kg group (Supplementary Table 1). No significant differences in OS, cGRFS, cGvHD, relapse, or NRM were observed between the two dose groups, although EFS showed a trend favoring 2.5 mg/kg (*p* = 0.071; Supplementary Fig. 5). In IPTW-weighted analyses, both 2.5 (HR 0.35; 95% CI 0.14–0.88; *p* = 0.025) and 5 mg/kg (HR 0.36; 95% CI 0.19–0.65; *p* < 0.001) were associated with reduced risk of cGvHD compared with non-ATG. A trend toward higher relapse was observed with ATG 5 mg/kg (HR 1.65; 95% CI 0.94–2.88; *p* = 0.077), whereas ATG 2.5 mg/kg showed a trend toward improved cGRFS (HR 0.49; 95% CI 0.22–1.10; *p* = 0.086), although neither reached statistical significance (Supplementary Fig. 6). Baseline absolute lymphocyte count (ALC) at ATG initiation was also evaluated as a potential predictor of ATG exposure, but showed no significant association with cGRFS (data not shown).

### Predictors of cGRFS

In the IPTW-weighted univariate analysis, the adverse cytogenetic risk group, higher HCT-CI (≥ 3), ATG use, and reduced intensity conditioning (RIC) were significantly associated with cGRFS (*p* < 0.1). In the IPTW-weighted multivariate analysis, adverse cytogenetic risk (HR 1.80; 95% CI 1.03–3.15; *p* = 0.039) and HCT-CI ≥ 3 (HR 7.79; 95% CI 3.36–18.06; *p* < 0.001) remained independent predictors of worse cGRFS. In contrast, the use of ATG at 2.5 mg/kg was significantly associated with improved cGRFS compared with non-ATG (HR 0.30; 95% CI 0.14–0.66; *p* = 0.002). RIC conditioning was also associated with improved cGRFS (HR 0.52; 95% CI 0.29–0.92; *p* = 0.026, Table 3). Interaction analyses indicated that the benefit of ATG on cGRFS was attenuated in adverse cytogenetics (*p* = 0.025), with no significant interactions for HCT-CI or conditioning intensity (Supplementary Table 3).

**Table 3:** Inverse probability of treatment weighting (IPTW)-weighted univariate and multivariate analyses for chronic GvHD-free relapse-free survival in the entire cohort

	Univariate analysis		Multivariate analysis	
	HR (95% CI)	*p*-value	HR (95% CI)	*p*-value
Age, ≥ 48 years	0.93 (0.63–1.38)	0.736	–	–
Sex, male	0.96 (0.64–1.42)	0.844	–	–
Cytogenetic risk, adverse	2.03 (1.17–3.51)	0.011	1.80 (1.03–3.15)	0.039
HCT-CI, ≥ 3	3.41 (1.42–8.15)	0.005	7.79 (3.36–18.06)	< 0.001
Dose of ATG (overall)	–	0.092	–	0.006
Non-ATG (reference)	1.00	–	1.00	–
ATG: 2.5 mg/kg	0.49 (0.22–1.10)	0.087	0.30 (0.14–0.66)	0.002
ATG: 5 mg/kg	0.67 (0.41–1.09)	0.113	0.71 (0.44–1.51)	0.171
Conditioning regimen, RIC	0.51 (0.27–0.97)	0.041	0.52 (0.29–0.92)	0.026
Infused CD34 + cell dose, ≥ 4.0 (x10^6^/kg)	1.20 (0.81–1.78)	0.360		
Total T cell count, ≥ 29.5 (x 10^7^/kg)	1.21 (0.82–1.79)	0.326	–	–
ABO matched donor	1.27 (0.85–1.90)	0.232	–	–
Sex-matched donor	0.93 (0.63–1.38)	0.744	–	–
Hospital	–	0.164	–	–
A (reference)	1.00	–	–	–
B	0.82 (0.44–1.52)	0.539	–	–
C	1.00 (0.42–2.08)	0.983	–	–

All models were weighted by IPTW based on a propensity score including cytogenetic risk, HCT-CI, conditioning regimen, and infused CD34⁺ cell dose. Variables with *p* < 0.10 in univariate analysis were included in the multivariate model. ALC (> 0.04 × 10⁹/L at ATG initiation) was additionally evaluated with multiple imputation, but was not significantly associated with cGRFS (HR 1.27; 95% CI 0.86–1.87; *p* = 0.225)

*HR* hazard ratio, *CI* confidence interval, *HCT-CI* hematopoietic cell transplantation-comorbidity index, *RIC* reduced intensity conditioning, *CD34+* cluster of differentiation 34-positive cells

## Discussion

In this multicenter retrospective study of AML patients undergoing MSD allo-HSCT in CR1, we found that ATG use reduced the incidence of cGvHD and improved cGRFS. These effects were consistent in both unadjusted and adjusted analyses using IPTW and propensity score matching. Our findings are consistent with prior reports showing that ATG reduces the risk of cGvHD and contributes to improved quality of life in long-term survivors [24–26].

In subgroup analyses, the benefit of ATG was evident among patients with intermediate-risk cytogenetics, where cGRFS improvement was primarily driven by reductions in cGvHD, without an increase in relapse or NRM. By contrast, in patients with adverse-risk cytogenetics, ATG was associated with higher relapse risk and inferior EFS, and interaction testing confirmed a significant interaction between ATG use and cytogenetic risk (*p* = 0.025). These findings suggest that the efficacy of ATG may be influenced by disease biology. One plausible explanation is that ATG, through in vivo T-cell depletion, may compromise GvL activity, which is particularly critical for disease control in biologically aggressive AML. While reduction in cGvHD improves long-term outcomes, this benefit may be offset if donor-derived antileukemic immunity is simultaneously weakened. Thus, the effect of ATG appears context-dependent: beneficial when relapse risk is moderate, but potentially detrimental when relapse risk predominates. Given the small number of patients with adverse cytogenetics (ATG *n* = 12; non-ATG *n* = 8), these findings should be considered exploratory and highlight the need for adequately powered prospective studies to validate and refine risk-adapted use of ATG.

With respect to dosing, both 2.5 and 5 mg/kg of ATG were effective in reducing cGvHD compared with non-ATG. Exploratory analyses suggested that 2.5 mg/kg may provide adequate prophylaxis without increasing relapse, whereas 5 mg/kg showed a trend toward higher relapse risk. Although neither association reached statistical significance, these divergent patterns raise the hypothesis that lower ATG exposure may achieve a more favorable balance between GvHD prevention and preservation of graft-versus-leukemia activity. Taken together with prior reports [11, 14, 15], these findings raise the possibility that 2.5 mg/kg may be sufficient in the MSD transplantation setting. Importantly, these results remained consistent after adjustment using IPTW and propensity score matching, indicating that the apparent adequacy of the reduced dose was not attributable to baseline imbalances between dose groups. Nevertheless, the limited number of patients in each dose subgroup (2.5 mg/kg, *n* = 17; 5 mg/kg, *n* = 41), precludes firm conclusions. Prospective studies incorporating pharmacokinetic monitoring, individualized dosing strategies, and biological correlates will be required to clarify the optimal ATG dose in MSD transplantation for AML.

Several studies have identified absolute lymphocyte count (ALC) at the start of ATG as a determinant of ATG exposure and a potential guide for individualized dosing [17–20, 27, 28]. In contrast, our analysis deliberately excluded ALC from the primary IPTW models due to substantial missingness and lack of a harmonizable counterpart in the non-ATG cohort. In sensitivity analyses with imputed ALC values, no significant association with cGRFS or other outcomes was observed. These findings suggest that while ALC-guided dosing may represent a useful framework, it captures a different dimension of ATG biology than the fixed-dose approach evaluated here. Larger prospective studies will be needed to reconcile these strategies and clarify whether ALC can refine ATG dosing in the context of disease-specific risk factors such as cytogenetics.

CMV reactivation occurred in a subset of patients but showed no significant association with relapse or NRM. Given the limited number of evaluable cases, these results should be interpreted with caution. Previous studies in AML have suggested that ATG may differentially affect CMV risk [8, 29–32]. we did not observe such an association, but integration of infectious outcomes in future studies may provide a more comprehensive assessment of the risk–benefit profile of ATG.

Several limitations should be acknowledged. First, the retrospective design may have introduced center-specific selection biases in ATG administration and dosing, although no significant inter-institutional outcome differences were observed. Second, subgroup analyses—particularly in adverse-risk cytogenetics and low-dose ATG—were constrained by small sample sizes, limiting statistical power. Third, the long enrollment period spanning two decades introduces potential era effects. Finally, the missingness of ALC data and the limited number of evaluable CMV reactivation events further constrained statistical power. These limitations highlight the need for larger, prospective, and biologically integrated studies to validate and extend our findings.

Taken together, our results should be regarded as hypothesis-generating and interpreted with appropriate caution. Nevertheless, they provide evidence that ATG can reduce chronic GvHD and improve cGRFS in AML patients undergoing MSD transplantation, while also suggesting that its benefit may be attenuated in adverse cytogenetic risk.

In conclusion, ATG use was associated with a lower incidence of chronic GvHD and improved cGRFS in AML patients undergoing matched sibling donor transplantation, with consistent results after IPTW adjustment. The principal benefit of ATG appears to lie in the prevention of chronic GvHD, thereby contributing to improved long-term transplant outcomes. However, this benefit was attenuated in patients with adverse cytogenetics. Exploratory dose analyses suggested a possible signal of improved cGRFS at 2.5 mg/kg and a potential increase in relapse risk at 5 mg/kg, but these findings require cautious interpretation given the limited sample size. Collectively, our study highlights the need for larger, prospective investigations to validate these observations and supports ongoing efforts to refine individualized, cytogenetic risk–adapted ATG strategies in AML allo-HSCT.

## Supplementary Information

Below is the link to the electronic supplementary material.

Supplementary Material 1 (DOCX 1.88 MB)

## Data Availability

No datasets were generated or analysed during the current study.
