# Supplementary material for: Cytogenetic risk–associated outcomes of antithymocyte globulin use in HLA-matched sibling transplantation for acute myeloid leukemia
Source: Ann Hematol. 2025 Oct 16;104(11):5995–6004. doi: 10.1007/s00277-025-06608-3 (PMC12672648; doi:10.1007/s00277-025-06608-3)
Supplement: Supplementary file 1 — Supplementary Material 1 (DOCX 1.88 MB) [file 277_2025_6608_MOESM1_ESM.docx]

**Cytogenetic Risk–Associated Outcomes of Antithymocyte Globulin Use in HLA-Matched Sibling Transplantation for Acute Myeloid Leukemia**

Mihee Kim*^1,2^, Ik-Chan Song*^3^, Seo-Yeon Ahn^1,2^, Ho Cheol Jang^1^, Jeong Suk Koh^3^, Chang-Hoon Lee^4^, Hyeoung-Joon Kim^1,2^, Ho-Young Yhim ‡^4^, Jae-Sook Ahn ‡^1,2^

^1^Hematology-Oncology, Chonnam National University Hwasun Hospital, Hwasun-gun, Jeollanam-do, Republic of Korea, ^2^Genomic Research Center for Hematopoietic Diseases, Chonnam National University Hwasun Hospital, Hwasun-gun, Jeollanam-do, Republic of Korea, ^3^Division of Hematology/Oncology, Department of Internal Medicine, Chungnam National University College of Medicine, Daejeon, Republic of Korea, ^4^Hematology-Oncology, Department of Internal Medicine, Jeonbuk National University Medical School, Jeonju, Republic of Korea

**Supplementary table 1. Balance summary of baseline covariates between anti-thymocyte globulin (ATG) and non-ATG groups before and after inverse probability of treatment weighting (IPTW)**

|  | Standardized Mean Difference (SMD) | |  |
| --- | --- | --- | --- |
| variable | Unadjusted SMD | Adjusted SMD | Balance threshold |
| Cytogenetic risk | 0.118 | 0.010 | Balanced, <0.100 |
| HCT-CI (≥3 vs < 3) | 0.057 | -0.055 | Balanced, <0.100 |
| Infused CD34+ cell dose (≥ 4 vs <4 x10^6^/kg) | -0.175 | 0.027 | Balanced, <0.100 |
| Conditioning regimen (MAC vs. RIC) | 0.147 | -0.057 | Balanced, <0.100 |

Absolute SMD < 0.100 was considered indicative of adequate balance.

Abbreviations: SMD, standardized mean difference; ATG, anti-thymocyte globulin; HCT-CI, hematopoietic cell transplantation-comorbidity index; CD34+, 34-positive cells; MAC, myeloablative conditioning; RIC, reduced-intensity conditioning

**Supplementary table 2. Baseline Characteristics of Patients according to anti-thymocyte globulin (ATG) dose (2.5mg/kg vs. 5mg/kg).**

|  | ATG: 2.5mg/kg  (n=17) | ATG: 5mg/kg  (n=41) | *p*-value |
| --- | --- | --- | --- |
| Age, years | 59.5 (18.0–70.0) | 52.0 (23.0–65.0) |  |
| ≥ 48 | 12 (70.6) | 26 (63.4) | 0.764 |
| < 48 | 5 (29.4) | 15 (36.6) |  |
| Time from diagnosis to HSCT, months | 5.7 (3.0–7.2) | 4.1 (2.9–19.3) | 0.170 |
| Sex, male | 8 (47.1) | 22 (53.7) | 0.775 |
| Cytogenetic risk |  |  | 0.478 |
| Intermediate | 14 (88.2) | 30 (75.6) |  |
| Adverse | 2 (11.8) | 10 (24.4) |  |
| ELN 2022 risk stratification | n = 17 | n = 38 | 0.396 |
| Favorable | 1 (5.9) | 1 (2.6) |  |
| Intermediate | 13 (76.5) | 24 (63.2) |  |
| Adverse | 3 (17.6) | 13 (34.2) |  |
| HCT-CI score |  |  | <0.001 ^a^ |
| 0 | 7 (41.2) | 36 (87.8) | 0.005 ^b^ |
| 1-2 | 6 (35.3) | 5 (12.2) |  |
| ≥3 | 4 (23.5) | 0 (0.0) |  |
| Conditioning regimen |  |  | 0.334 |
| MAC | 11 (64.7) | 32 (78.0) |  |
| RIC | 6 (35.3) | 9 (22.0) |  |
| ABO matched donor | 6 (35.3) | 30 (73.2) | 0.015 |
| Sex-matched donor | 9 (52.9) | 18 (43.9) | 0.574 |
| Infused CD34+ cell dose (median, x10^6^/kg) | 4.83 (2.28–13.18) | 3.58 (0.98–13.40) |  |
| ≥ 4.0 | 11 | 13 | 0.038 |
| < 4.0 | 6 | 28 |  |
| Total T cell count (median, x 10^7^/kg) | 23.86 (13.53–30.3) | 29.12 (14.73–56.3) |  |
| ≥ 29.5 | 2 (11.8) | 19 (46.3) | 0.030 |
| < 29.5 | 15 (88.2) | 22 (53.7) |  |
| ALC on the day of ATG initiation  (median, x 10^9^/L) | n = 5  0.02 (0–0.04) | n = 41  0.02 (0–0.64) |  |
| > 0.04 | 0 | 12 (29.3) | 0.306 |
| ≤ 0.04 | 5 (100) | 29 (70.7) |  |
| Duration of follow-up, months | 32.1 (5.8–70.4) | 69.3 (5.2–100.3) | 0.009 |

Values are presented as number (%) or median (range), unless otherwise indicated.

^a^ Overall comparison across the three groups.

^b^ Comparison between HCT-CI ≥3 vs <3.

Abbreviations: ATG, antithymocyte globulin; HSCT, hematopoietic stem cell transplantation; ELN, European LeukemiaNet; HCT-CI, hematopoietic cell transplantation-comorbidity index; MAC, myeloablative conditioning; RIC, reduced-intensity conditioning; CD34+, 34-positive cells; ALC, absolute lymphocyte count.

**Supplementary table 3. Interaction effects of antithymocyte globulin (ATG) with clinical covariates on chronic graft-versus-host disease (cGvHD)-free relapse-free survival (cGRFS).**

| Variable (Interaction term) | HR (95% CI) | *P* value |
| --- | --- | --- |
| ATG * Cytogenetic risk | 3.17 (1.12-8.97) | 0.029 |
| ATG * HCT-CI | 0.43 (0.04-4.42) | 0.477 |
| ATG * Conditioning regimen | 0.85 (0.27-2.66) | 0.790 |

Interaction terms reflect whether the effect of ATG use on cGRFS varies according to cytogenetic risk (adverse vs. intermediate), HCT-CI (≥3 vs. <3), or conditioning regimen (myeloablative conditioning vs. reduced-intensity conditioning)

Abbreviations: ATG, antithymocyte globulin; HCT-CI, hematopoietic cell transplantation-comorbidity index; MAC, myeloablative conditioning; RIC, reduced-intensity conditioning; CD34+, 34-positive cells.

**
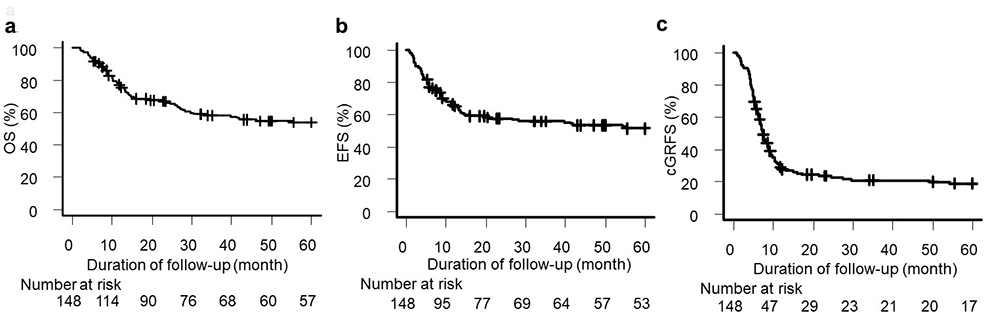
**

**Supplementary Fig.1** Outcomes in the Entire Cohort (a) Overall survival (OS), (b) event-free survival (EFS), and (c) chronic graft-versus-host disease (cGvHD)-free relapse-free survival (cGRFS) in all patients

**
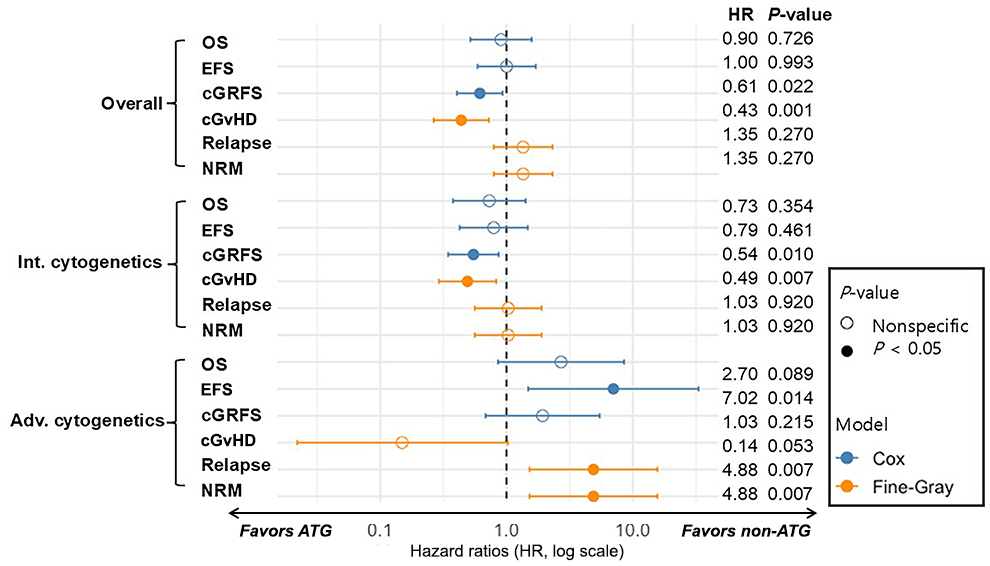
**

**Supplementary Fig.2** Forest plot of inverse probability of treatment weighting (IPTW)-weighted hazard ratios comparing ATG versus non-ATG for overall survival (OS), event-free survival (EFS), chronic graft-versus-host disease (cGvHD)-free relapse-free survival (cGRFS), cumulative incidence of chronic GvHD, relapse, and non-relapse mortality (NRM) in the overall cohort (top), and stratified by cytogenetic risk (intermediate-risk vs. adverse-risk, below). Hazard ratios <1.0 favor ATG, >1.0 favor non-ATG. Multivariable Cox models and Fine–Gray competing risk models were adjusted using inverse probability of treatment weighting. Bold circles indicate statistically significant hazard ratios (*p* < 0.05).

**
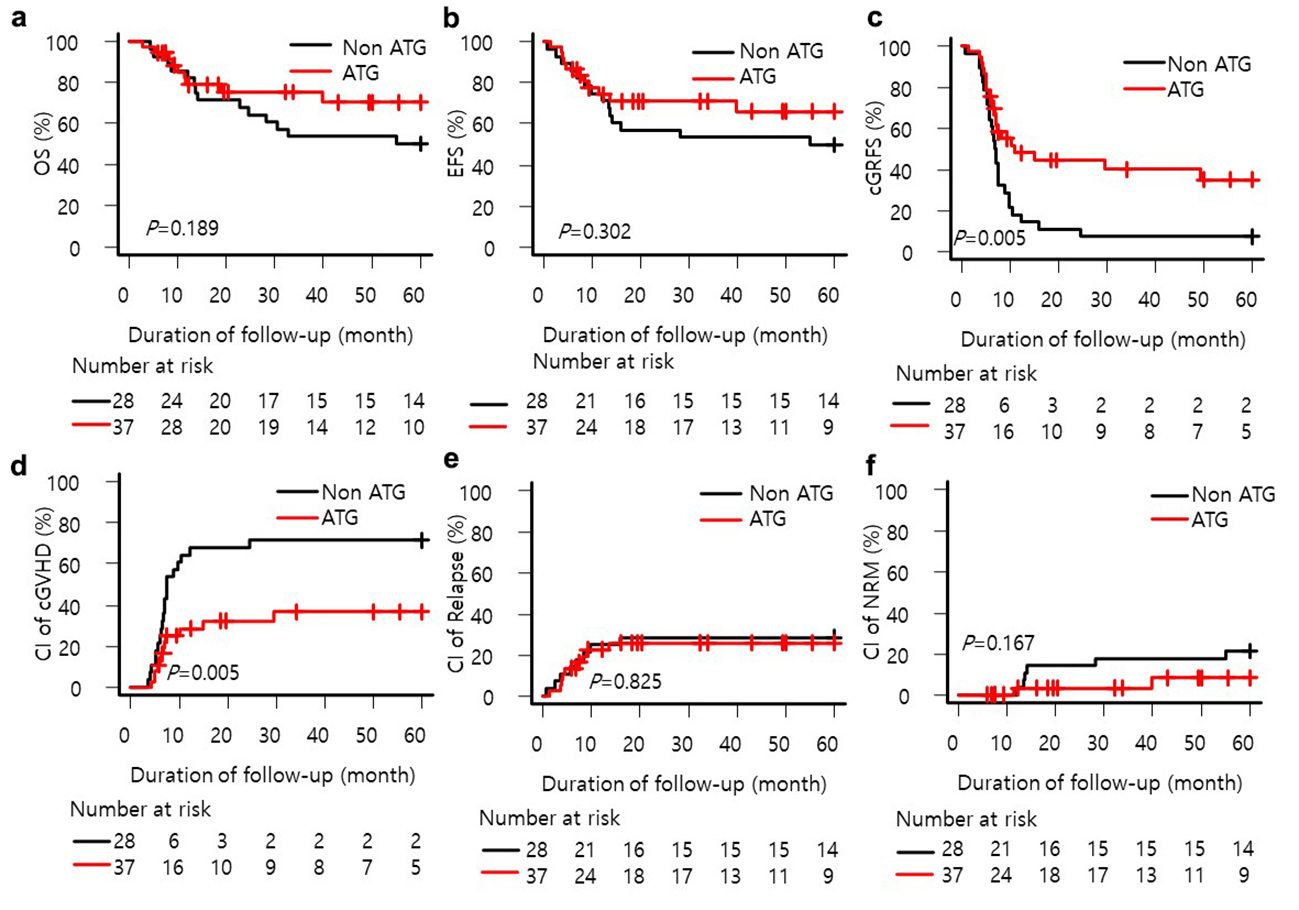
Supplementary Fig.3** Transplantation outcomes in patients with ELN-2022 Intermediate-risk AML, stratified by antithymocyte globulin (ATG) administration (unadjusted) (a) Overall survival (OS), (b) event-free survival (EFS), (c) chronic graft-versus-host disease (cGvHD)-free relapse-free survival (cGRFS), (d) cumulative incidence (CI) of cGvHD, (e) cumulative incidence of relapse, and (f) cumulative incidence of non-relapse mortality (NRM). ATG use was associated with improved cGRFS (IPTW-adjusted HR 0.49, 95% CI 0.28–0.89, *p* = 0.019) and reduced risk of cGvHD (HR 0.40, 95% CI 0.21–0.75, *p* = 0.005), while no significant differences were observed in OS (HR 0.57, 95% CI 0.24–1.39, *p* = 0.217), EFS (HR 0.70, 95% CI 0.31–1.59, *p* = 0.387), relapse (HR 0.98, 95% CI 0.43–2.27, *p* = 0.970), or NRM (HR 0.98, 95% CI 0.43–2.27, *p* = 0.970).

**
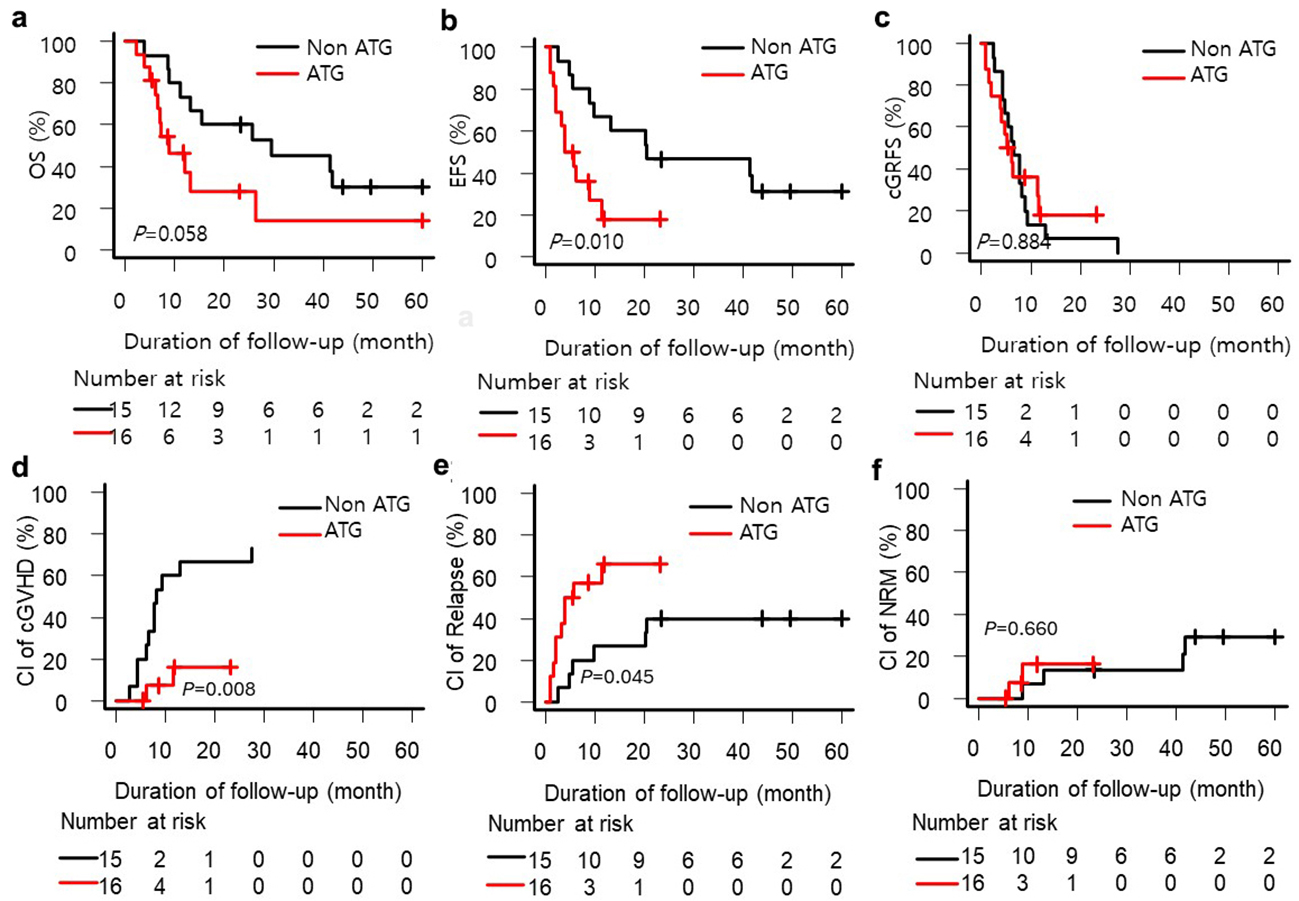
Supplementary Fig.4** Transplantation outcomes in patients with ELN-2022 adverse-risk AML according to antithymocyte globulin (ATG) administration (unadjusted) (a) Overall survival (OS), (b) event-free survival (EFS), (c) chronic graft versus host disease (cGvHD)-free relapse-free survival (cGRFS), (d) cumulative incidence (CI) of cGvHD, (e) cumulative incidence of relapse, and (f) cumulative incidence of non-relapse mortality (NRM)

ATG use was associated with a significantly lower risk of cGvHD (IPTW-adjusted HR 0.20, 95% CI 0.05–0.82, p = 0.026), while no significant differences were found in cGRFS (HR 0.91, 95% CI 0.35–2.38, p = 0.851), OS (HR 1.93, 95% CI 0.73–5.11, p = 0.188), EFS (HR 2.44, 95% CI 0.86–6.92, p = 0.094), relapse (HR 2.36, 95% CI 0.90–6.22, p = 0.081), or NRM (HR 2.36, 95% CI 0.90–6.22, p = 0.081).

**
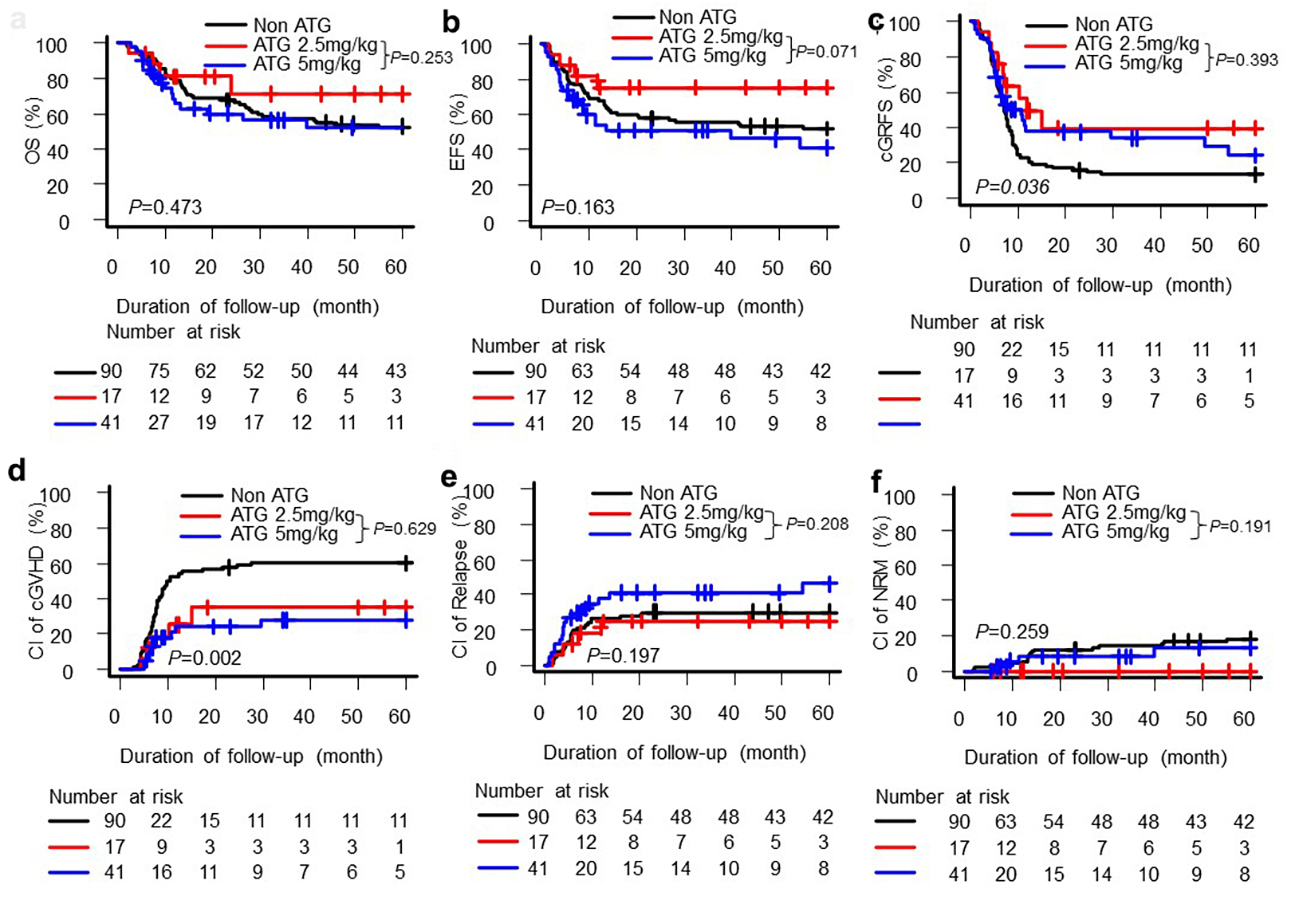
**

**Supplementary Fig.5** Transplantation outcomes according to antithymocyte globulin (ATG) dosing strategy: non-ATG, ATG 2.5mg/kg, and ATG 5mg/kg (a) Overall survival (OS), (b) event-free survival (EFS), (c) chronic graft-versus-host disease (cGvHD)-free relapse-free survival (cGRFS), (d) cumulative incidence (CI) of cGvHD, (e) cumulative incidence of relapse, and (f) cumulative incidence of non-relapse mortality (NRM).

**
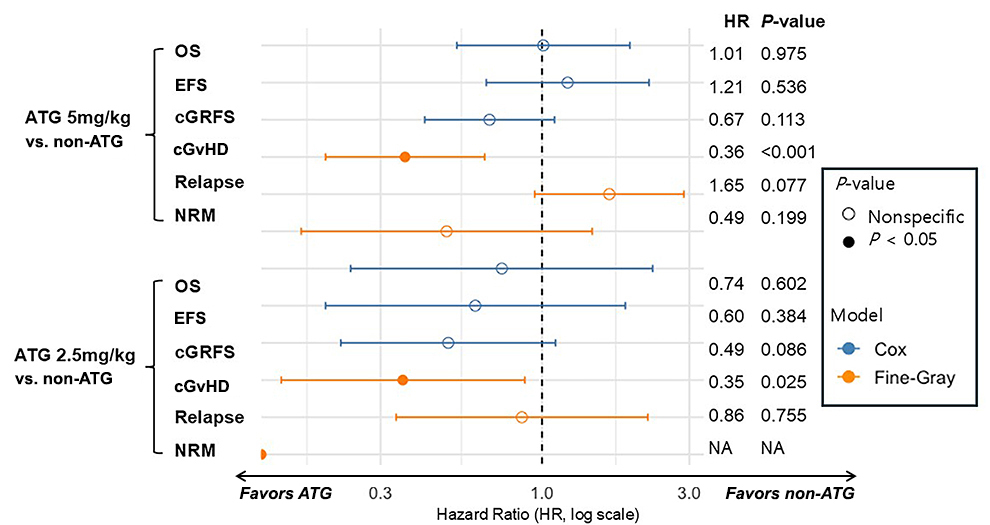
**

**Supplementary Fig.6** Inverse probability of treatment weighting (IPTW)-weighted forest plot of transplantation outcomes according to ATG dose. Hazard ratios for overall survival (OS), event-free survival (EFS), chronic graft-versus-host disease (cGvHD)-free relapse-free survival (cGRFS), cumulative incidence of chronic GVHD, relapse, and non-relapse mortality (NRM) are shown for patients receiving ATG 5 mg/kg (top) or 2.5 mg/kg (below), each compared with the non-ATG group as the reference. Hazard ratios <1.0 favor ATG, >1.0 favor non-ATG. Multivariable Cox models and Fine–Gray competing risk models were adjusted using inverse probability of treatment weighting. Bold circles indicate statistically significant hazard ratios (*p* < 0.05).
